# Allometric Growth Patterns and Phenotypic Plasticity Indices of Different Grades of Annual *Pinus yunnanensis* Franch. Seedlings at Different Growth Stages

**DOI:** 10.3390/biology15131008

**Published:** 2026-06-25

**Authors:** Pengrui Wang, Zhuangyue Lu, Yulan Xu, Nianhui Cai

**Affiliations:** 1 The Key Laboratory of Forest Resources Conservation and Utilization in the Southwest Mountains of China Ministry of Education, Southwest Forestry University, Kunming 650224, China; 18387876686@163.com (P.W.); luzhuangyue@swfu.edu.cn (Z.L.); xuyulan@swfu.edu.cn (Y.X.); 2 The Key Laboratory of National Forestry and Grassland Administration on Biodiversity Conservation in Southwest China, Southwest Forestry University, Kunming 650224, China

**Keywords:** *Pinus yunnanensis* Franch., seedling quality, growth trend, morphological trait

## Abstract

*Pinus yunnanensis* Franch. is a native pioneer and economically important tree in Yunnan Province in China. In this study, over 1400 annual seedlings were used. Following national or regional official seedling quality standards, seedlings were classified into three grades, namely Grade I, Grade II, and Grade III by using mean ± 1/2 standard deviation method according to the height of seedlings (H ± 1/2σ). Morphological traits including seedling height, ground-line diameter, root length, and root average diameter were measured from September 2022 to December 2023 for each grade. A power-law allometric growth model was constructed, and the standardized major axis method was used to analyze the allometric relationships between plant height and ground-line diameter as well as between root length and root average diameter. The results showed that higher grade seedlings exhibited stronger synergistic plasticity, accelerating allometric growth and enhancing phenotypic plasticity. A significant positive correlation was found between plant height and ground-line diameter growth rates, with ground-line diameter showing greater plasticity. Grade I seedlings demonstrated clear advantages, with mean allometric rates of 0.5860 for plant height versus ground-line diameter and 1.6315 for root length versus root system. The phenotypic plasticity index for ground-line diameter was high across all three grades, but actual thickening varied by grade due to different initial diameters, with Grade I and II seedlings growing much more than Grade III. For plant height, the index ranged from 0.3 to 0.8, with values of 0.6–0.7 for Grade I, 0.3–0.7 for Grade II, and 0.6–0.8 for Grade III. These findings provide a scientific basis for evaluating seedling quality, breeding, reproduction, and improving survival and growth in later-stage afforestation.

## Simple Summary

*Pinus yunnanensis* Franch. is an important native tree in Southwest China, but its seedlings often grow slowly. We divided annual seedlings into three grades using national standards. We measured plant height, ground-line diameter, and roots, calculated phenotypic plasticity index. The three grades differed greatly. Grade I grew fastest and adjusted quickly. Grade II started slow but sped up. Grade III grew worst with large variation. The biggest differences occurred in summer and autumn. We suggested early grading and different water and fertilizer management in those seasons. This will increase high-quality seedlings, reduce waste, and improve survival and forest uniformity.

## 1. Introduction

*Pinus yunnanensis* Franch. is a native tree species and a pioneer species in the southwest region, and is also the main economic species in Yunnan province [1]. Moreover, *P. yunnanensis* is known for its high ecological adaptability and strong natural regeneration potential, and is primarily used for seedling cultivation and afforestation [2]. However, *P. yunnanensis* grows extremely slowly after afforestation, and its seedlings mainly remain in a growth-inhibited stage in practical forestry production [3]. The long nursery period significantly reduces afforestation efficiency, making it difficult to fully realize the ecological value of this species [3]. In recent years, most *P. yunnanensis* forests have become pure forests, characterized by a high proportion of individuals, exhibiting topographic morphology, bending and distortion, and dwarfing [4]. In addition, the problem of genetic degradation in *P. yunnanensis* is becoming increasingly serious [5]. Asexual reproduction has two main advantages: offspring traits are stable, and the reproduction cycle is short [2]. This makes it a good solution to the problems of using seed-grown *P. yunnanensis* seedlings for planting forests [3]. It overcomes issues like low flower and seed production and large differences in seedling appearance [6]. High-quality and abundant nursery cultivation provides a solid foundation for successful asexual reproduction [7]. The key to seedling grades is determining the evaluation index of seedling quality [8]. The study of seedling grades plays a good role in promoting the cultivation of seedlings [9]. Researchers have found that, in production practice, plant height, ground-line diameter, and root system were relatively direct indicators of seedling grading, and were also measures of the leading indicators of seedling quality [10]. Currently, there are many studies on seedling grades. For example, researchers have studied the seedling grades and growth of *Zanthoxylum nitidum* by clustering the grading critical point method [11]. In addition, researchers combined afforestation experiments and cluster analysis to study the seedlings of *Illicium verum* in Gannan and its grading standards [12]. Further study on seedling grades of annual *P. yunnanensis* is now needed. Besides, the growth and development of annual *P. yunnanensis* at the seedling stage affects the growth of later stages and even the whole life [13]. In summary, the research on the seedling grades of annual *P. yunnanensis* is not only helpful for the growth detection of annual *P. yunnanensis*, but also can improve the benefits of forest breeding and late afforestation.

Moreover, determining the index that can accurately reflect the quality of seedlings and is convenient for practical application is one of the essential research topics in seedling grades [14]. The evaluation of seedling quality is primarily based on morphological indexes, such as seedling height, ground-line diameter, and root condition [8]. These morphological indices reflect the seedling’s morphological characteristics and growth trend [8]. Seedling height and ground-line diameter are the most common parameters for assessing seedling quality [15]. Recently, root morphological indexes have also been studied more deeply [15]. Seedling height is the most intuitive and easily measured morphological indicator, and even without equipment, people can visually observe the height of the plant seedlings [16]. Due to the influence of site conditions, selecting seedlings with a large initial height is beneficial for improving the afforestation effect [17]. The research showed that the initial seedling height, specific root volume and lateral root number of *Quercus rubra* and *Q. alba* were better predictors of afforestation effect under weed control [15]. Ground-line diameter is defined as the stem diameter at the soil surface, and largely determined by root system size and directly influences seedling survival and early post-planting growth [16]. Some studies suggested that ground-line diameter is the best predictor of afforestation. For example, the root average diameter, main root length and lateral root number of bare root seedlings of *Q. rubra* were significantly positively correlated with the growth status after 2 years of afforestation [18]. A key component of plant anatomy is the root system, and it is the key to determining the survival of seedlings after afforestation and the growth of young forest [16]. Seedlings of different grades can be distinguished by their distinct morphological traits and developmental stages [19]. After calculating the mean ± standard deviation of the seedlings, the researchers used cluster analysis method to divide the seedlings into Grade I, II and III according to the needs of seedling production practice [12]. Grade I and II seedlings can be used for afforestation, and Grade III seedlings are unqualified seedlings and need to be further cultivated [14]. The *Oplopanaxs elatus* showed that the critical value of root length of Grade I seedlings was greater than that of Grade II seedlings and that of Grade III seedlings [20]. Therefore, the quality of seedlings is one of the important factors affecting the effectiveness of afforestation; quality directly determines the level of afforestation survival rate and stand growth. At present, the seedling quality of annual *P. yunnanensis* still needs further study. The scientific and accurate detection of the seedling quality in annual *P. yunnanensis* contributes significantly to the success of afforestation projects.

Allometric growth refers to the disproportionate growth relationship between the relative growth rates of two traits in an organism [21]. Moreover, allometric growth, also called dependent growth, describes the nonlinear quantitative relationship between the size of an organism and other properties [22]. Researchers can use them to predict and study organ allometric relationships and to predict and determine plant traits that are difficult to measure directly [23]. Therefore, allometric analysis is an effective method to study the change of plant growth and dry matter with time [24]. In recent years, there have been many advances in allometric growth. For example, researchers studied *Loropetalum chinense* shrubs of different stem diameters and from different places [25]. They found that how leaves grew relative to stems and roots followed the allometric distribution hypothesis [25]. In addition, a study of 40 herbaceous plants showed a significant allometric relationship between transpiration, leaf biomass, and body size [26]. Phenotypic plasticity is how plants change their traits as they grow, and it helps them respond to environmental conditions [27]. This ability is the visible basis of how plants genetically adapt. Sometimes, differences in appearance between species are simply because plants differ in size or age [28]. Allometric analysis can help us separate the effects of the environment from the effects of individual plant growth [28]. By studying how seedlings grow allometrically using the phenotypic plasticity index, we can better understand plant phenotypic plasticity and how plants adapt their growth to the environment [29]. However, the allometric growth of different seedling grades across different dates remains unclear. To address this gap, we used the mean ± 1/2 standard deviation method based on morphological traits to classify annual *P. yunnanensis* seedlings into Grades I, II, and III. Seedling height, ground-line diameter, and root-related indicators were measured on different dates to evaluate seedling quality and analyze allometric growth among grades. Based on previous allometric analysis of biomass allocation [21], we hypothesized that over time, seedling height and ground-line diameter will show a significant positive correlation across grades. Phenotypic plasticity indices and growth trends are expected to differ significantly among grades, with Grade I seedlings likely exhibiting the highest growth rate.

Previous researchers have studied the allometric growth of *P. yunnanensis* by setting different shading levels and measuring biomass [30], but it is not clear how to study the allometric growth of *P. yunnanensis* by measuring the morphological characteristics of *P. yunnanensis* at different time points. Therefore, according to the national and regional seedling quality standards, the seedlings of *P. yunnanensis* were divided into three grades in this study to explore the allometric growth pattern of *P. yunnanensis* seedlings on different dates. By measuring morphological indicators such as plant height, ground-line diameter, and root system traits, the phenotypic plasticity index was used to analyze the morphological characteristics, growth trends, and growth rates of seedlings of different grades at various time points. This study classified *P. yunnanensis* seedlings into three grades based on national and local quality standards. We measured their height, ground-line diameter, and roots at different times, and also calculated the phenotypic plasticity index to observe how they grew and changed. By comparing the grades, we wanted to understand how the seedlings adapt and what growth strategies they use. Our goal is to give practical advice on early grading and different care for each grade, and to support better nursery practices. This will help more trees survive after planting and make forests healthier.

## 2. Materials and Methods

### 2.1. Location for the Research

**Figure 1:**
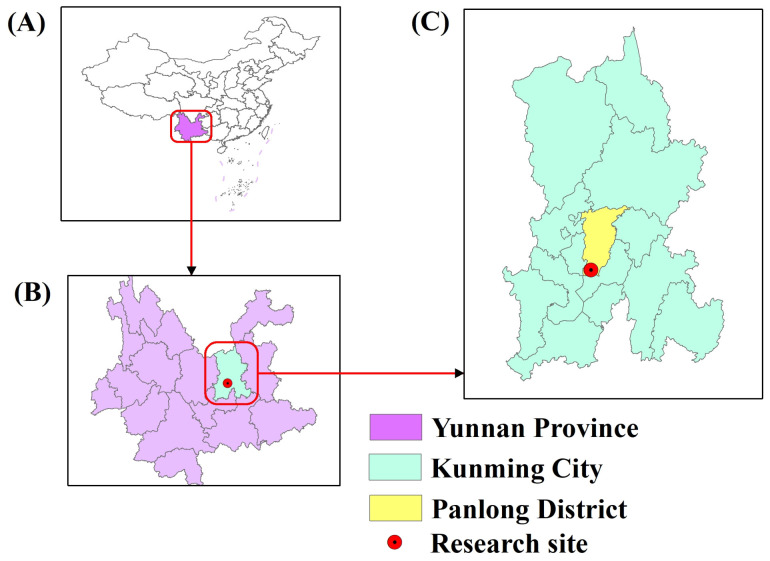
The general area of the study’s experimental site. (**A**) Yunnan Province in China; (**B**) Kunming City in Yunnan Province; (**C**) Research site located in Panlong District in Kunming City.

The experimental work was performed in Kunming (25°04′00″ N 102°45′40″ E), a subtropical Southwest Forestry University monsoon at an altitude of about 1945 m (Figure 1). The climate was characterized by an average annual relative humidity of 68.2%, with annual precipitation ranging from 700 to 1100 mm, predominantly concentrated from May to October. The average annual sunshine duration was about 2446 h, and the area’s climate was characterized by a mean annual evaporation of 1856 mm, and the soil was low-acidity phosphorus red soil.

### 2.2. Sources of Seeds

Seed samples of *P. yunnanensis* were obtained from composite seed lots at the Clonal Seed Orchard located in Midu, Yunnan. The mother trees chosen for sampling were fully mature and demonstrated strong fruiting ability. The seeds were washed with clear water, first soaked in 50 °C warm water for 24 h, and then planted after soaking in clear water for 24 h. Experimental seedling substrate quality compost soil was selected from a domestic substrate company. It was evenly mixed with Southwest Forestry University with a substrate formulated from humus soil and red soil in a 3:1 ratio. Then, plastic pots of about 13 cm in height, about 14.5 cm in diameter, and about 15.5 cm in diameter were selected in the Southwest Forestry University nursery for cultivation, with a volume of about 306.9 cm^3^. All seedlings of *P. yunnanensis* were grown under completely randomized and consistent nursery conditions, where environmental factors were kept the same. The key of site characteristics included annual average temperature of 16.2 °C, extreme temperature values of 34.5 °C and −6.8 °C, average relative humidity of 70%, and elevation of 1900–2000 m. Mountain red soil fertility was moderate with a pH of 6.0. Seeds were soaked in 0.5% KMnO_4_ for 30 min, and after being rinsed and disinfected with distilled water, were subjected to 24 h of hydration in 50 °C water before culture.

### 2.3. Classification of Seedlings

**Figure 2:**
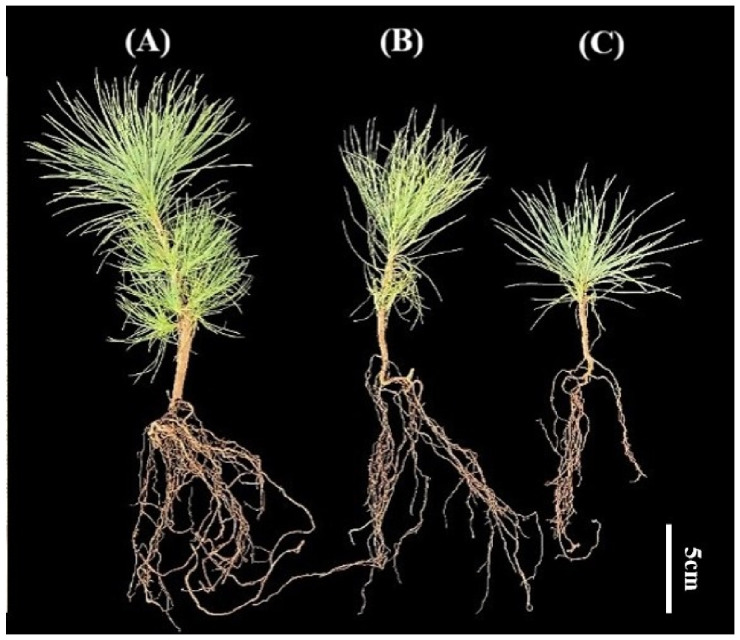
The different grades of *P. yunnanensis* seedlings. (**A**) The Grade I of *P. yunnanensis* seedlings; (**B**) The Grade II of *P. yunnanensis* seedlings; (**C**) The Grade III of *P. yunnanensis* seedlings.

Seedling height (H) and stem diameter at ground level (D) were typically used as quality indicators for seedling grading. However, due to strong correlations among various quality indicators, the use of multiple metrics can lead to information redundancy. Seedling grading criteria should not only be scientifically sound but also align with production practices and practical applications, emphasizing applicability and operability. Therefore, this study used seedling height as the sole quality indicator for grading. Following the “mean ± 1/2 standard deviation” as the grading criterion, seedlings were categorized into three grades: Grade I, Grade II, and Grade III (Figure 2).

With reference to national and regional seedling quality standards, the calculation formulas for grade definition were as follows: [31]

G = H ± 1/2 σ(1)

Here, G represented the grading standard, H represented the mean seedling height, and σ represented the population standard deviation of height.

Seedling quality was measured by height, and grades were based on that. The national standard (LY/T 1950-2011) [32] provided the rules for quality indicators and grading. The standard formula for the size range of Grade I seedlings was as follows:

I ≥ H ± 1/2 σ(2)

The standard formula for the size range of Grade II seedlings was as follows:

H − 1/2σ ≤ II < H + 1/2σ(3)

And the standard formula for the size range of Grade III seedlings was as follows:

III < H − 1/2σ(4)

The sample sizes for each grade were as follows: Grade I had a sample size of 468, Grade II had a sample size of 569, and Grade III had a sample size of 411. After grading, all seedlings from different grades were grown under uniform conditions for later experiments.

### 2.4. The Measurement of the Correlative Index of the Seedling

#### 2.4.1. Measurement of Seedling Height and Ground-Line Diameter

Based on the seasonal growth characteristics of annual *P. yunnanensis* and the climatic conditions in the study area, we determined the growth index of 3 grades of seedlings from September 2022 to 2023 December. The height of the plant was measured with a ruler accurate to 0.01 cm. Seedling ground-line diameter was measured with vernier calipers accurate to 0.01 mm.

#### 2.4.2. Determination of Root System Index of Seedlings

There were 3 biological replicates of 9 seedlings in each treatment, and 3 technical replicates of each seedling were conducted by measuring the root length and the root average diameter of multiple roots. This design was commonly used in root studies, Studies showed the soil core method needed only 5 to 12 samples for root length and volume under 90 percent confidence and 80 percent accuracy, and 3 to 10 samples to cover 80 to 95 percent of root diameter classes. Thus, root studies required far fewer than *n* ≥ 30 samples, supporting the design of 9 plants per group as three replicates with three plants each as methodologically valid [33]. We used the Epson root scanner to scan the root system and then used WinRHIZO Basic software to analyze it. We counted the number of roots in September and December 2022, and the number of roots in September and December 2022, root length and root average diameter of annual *P. yunnanensis* seedlings 2023 in March, June, September and December, the speed and trend of root allometric growth of different grades of seedlings were analyzed by measuring the growth relationship between root length and root average diameter.

#### 2.4.3. Phenotypic Plasticity Index Measurement

The study utilized annual *P. yunnanensis* seedlings representing different competitive grades planted in annual flowerpots. After testing the height, ground-line diameter and root system of different grades of annual *P. yunnanensis* seedlings on different dates, the maximum and minimum values of each variable were counted, and the phenotypic plasticity index was finally calculated. The phenotypic plasticity exponent is PI = (Max − Min) ÷ Max. In the formula, Max and Min are defined as the maximum and minimum mean values of the variables, respectively.

### 2.5. Construction and Analysis of Allometric Growth Model

Allometric growth described a nonlinear quantitative relationship [34], usually expressed as a power function (5):

*Y* = *a*
*x*^*b*^, (5)

To convert linearly to:

*log Y* = *b*
*log x* + *log a* (6)

In the Formula (6): *Y* was the height (cm) or root length (mm) of the plant; *x* was the ground-line diameter (cm) or root average diameter (mm); *log a* was the intercept of the trait relationship; *b* was the slope, the allometric index between grades, and If *b* was not significantly different from 1, the growth type was isometric growth; if it was significantly different, the growth type was allometric growth.

By using the standardized principal axis estimation method of SMATR package version 3.4-8 in R software [35], the parameter estimation of the allometric equations of height and ground-line diameter and root length-root average diameter of different grades of annual *P. yunnanensis* seedlings on different dates was obtained, and the slope *b* was obtained, then compared the differences between the slopes and between the slopes and 1.0. If the slope *b* of different grades was significantly different, it indicated that the allometric relationship has changed. If there was no significant difference between the slopes, given the common slope, it was necessary to further compare whether their intercepts were the same, and further multiple comparisons, that was to determine whether they had the same linear fitting axis. Allometric index (*b*) and intercept (log *a*), intercept drift test, and analysis of variance were calculated by the SMATR package of R software. In addition, the allometric growth level of each equation can also be evaluated by the *p* value. A significance level of *p* < 0.05 was interpreted as evidence of allometric growth, whereas *p* ≥ 0.05 was classified as isokinetic growth.

### 2.6. Data Processing and Analysis

We used Excel to record the growth data of different grades of annual *P. yunnanensis* seedlings on different dates. Secondly, we used IBM SPSS Statistics 27 software to count the number of different grades of annual *P. yunnanensis* seedlings. Meanwhile, we used SMATR package version 3.4-8 in R software [35] to analyze the linear relationship between seedling height and ground-line diameter allometric growth of annual *P. yunnanensis* seedlings on different dates, and the relationship between seedling height and ground-line diameter allometric growth was analyzed, and the linear relationship between root length and root average diameter of allometric growth. Besides, the linear model of the allometric growth equation was constructed, and the growth trend and rate of different grades of annual *P. yunnanensis* seedlings on different dates were observed. Finally, we used the Origin 2025 software to draw the allometric images of seedling height, ground-line diameter, and root system of different grades of annual *P. yunnanensis* under different dates, which can more intuitively show the growth trend of different grades of annual *P. yunnanensis*, so as to reveal the adaptation mechanism of different grades of annual *P. yunnanensis* seedlings with time.

## 3. Results

### 3.1. Allometric Growth of Height and Ground-Line Diameter of Seedlings of Different Grades

Since there was almost no change in ground diameter, seedling height, and biomass accumulation in January, the growth rhythm characteristics of *P. yunanensis* largely overlap with those of December and February. Therefore, to focus resources on monitoring the active growth period from March to November and the transition periods in December and February before and after dormancy, January was not included as an independent sampling time point. As shown in Table 1, the analysis of growth data from September 2022 to December of the 2023 year for three different grades found that all models had a *p* value of 0.000, indicating that growth data from September 2022 to December of the year 2022 were not sensitive to environmental factors, the results showed that the growth type between seedling height and ground-line diameter was allometric growth, and the growth relationship was highly significant, but the *R*^2^ values were generally low, most of which were less than 0.3, indicating that the model had certain limitations, may be influenced by other factors. The slope of Grade I increased from 0.4337 to 0.7441, while the intercept decreased from 0.7289 to 0.2688. The *R*^2^ value decreased continuously, indicating that the growth regulation mechanism may be unstable. Grade II in 2023 had its best midterm fit with a February *R*^2^ peak of 0.322, while Grade III was relatively stable with intercepts ranging from 0.4946 to 0.5833 and a less volatile slope. In conclusion, the growth pattern of Grade I changed most dramatically, Grade II fitted well in a specific period, and the growth trend of Grade III was relatively stable.

**Figure 3:**
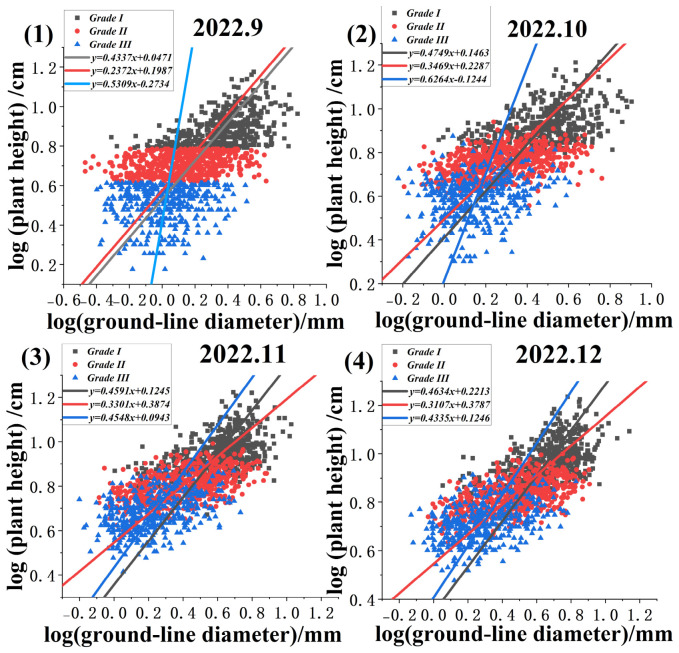
Linear function image of allometric growth equation between height and ground−line diameter of different grades of annual *P. yunnanensis* seedlings on different dates of (**1**) September 2022, (**2**) October 2022, (**3**) November 2022, (**4**) December 2022.

Figure 3 illustrated the allometric equation linear function image of seedling height and ground-line diameter from September to December 2022. From September to December in 2022, after log-transformed data processing, there was a significant linear positive correlation between ground-line diameter and seedling height of Grade I, II and III seedlings, but the regression slope and intercept of each grade changed significantly with month and grade. From September to October, the slope of Grade I seedlings reached 0.6264, and the growth efficiency was better than that of Grade II and III seedlings. After October, the slope of Grade I seedlings decreased to 0.4591–0.4749, and the growth efficiency of Grade II and III seedlings was higher than that of Grade III seedlings, The most rapid increase in seedling height was found to occur in the initial phase, but the growth efficiency decreased month by month, and the growth rate showed a trend of “first fast and then slow”. The lowest slope of Grade II seedlings was 0.2372 in September, but it rapidly increased to 0.4335–0.4634 after November. The growth efficiency of Grade II seedlings increased significantly in the late stage, and the growth rate showed an accelerated catch-up of “first suppression and then rise”. The lowest slope of Grade III seedlings decreased from 0.3469 to 0.3301 in September and October, and the highest intercept was 0.3874 in December, which showed that the difference between individuals was enlarged in the later stage, indicating that the driving effect of ground-line diameter on seedling height was the weakest, and the height of Grade III seedlings was the highest, the growth rate maintained at a low level and slowed down slightly, and the differentiation between individuals increased. In general, the growth efficiency of Grade I seedlings was always high, the growth efficiency of Grade II seedlings was accelerated in the middle and late stages, and the growth correlation of Grade III seedlings was the weakest in the late stage. The differences between grades tended to converge over time but still existed.

**Figure 4:**
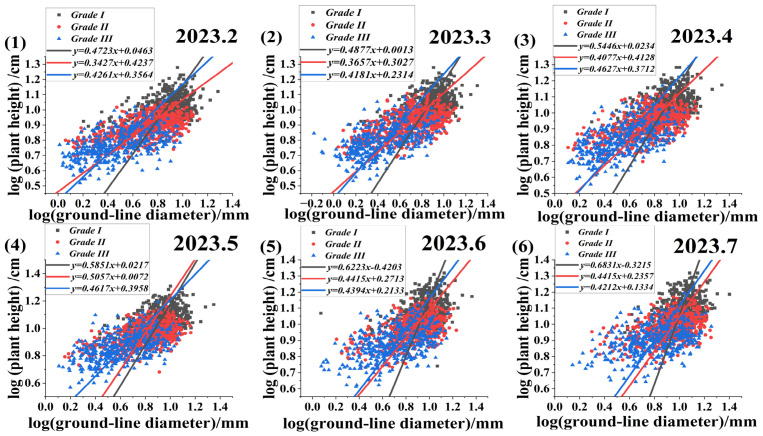
Linear function image of allometric growth equation between height and ground−line diameter of different grades of annual *P. yunnanensis* seedlings on different dates of (**1**) February 2023, (**2**) March 2023, (**3**) April 2023, (**4**) May 2023, (**5**) June 2023, (**6**) July 2023.

Figure 4 illustrated the plot of the linear function of the allometric equation for height and ground-line diameter from February to July 2023. From February to July, the 2023 ground-line diameter and height of Grade I, II and III seedlings maintained a significant linear relationship after log-transformed data processing. However, the evolution of slope and intercept showed a “summer acceleration” feature that was completely different from that in 2022: after the slope of the Grade I seedlings decreased slowly from 0.5446 in February to 0.4723 in April, it quickly rebounded to 0.5851–0.6831 in June and July, and the slope of Grade I seedlings increased from 0.5446 in February to 0.4723 in April. The growth efficiency changed from stable to strong, and the growth rate showed a secondary acceleration of “stable first and then fast”. The slope of the Grade II seedlings was the lowest of 0.4077 in February, and continued to rise to 0.4415–0.4627 from March to May, and further increased to 0.5057 in July. The growth rate was accelerated month by month, and the gap with the Grade I seedlings was narrowing. The slope of the Grade III seedlings was always the lowest, which was 0.3657 in February, the growth rate increased slightly to 0.4261 in April and then decreased to 0.4212 in July, and the intercept was 0.3027–0.4237, indicating that the driving efficiency of ground-line diameter on seedling height was low. Although the growth rate was briefly boosted in April, it later slowed down again, interindividual differentiation continues to expand. In general, the slope and growth rate of Grade I and Grade II seedlings showed a synchronous jump after summer, the dominance of Grade I seedlings was reamplified, the efficiency of Grade III seedlings was low and the difference was aggravated throughout the year, and the differentiation between grades reached the maximum in summer.

**Figure 5:**
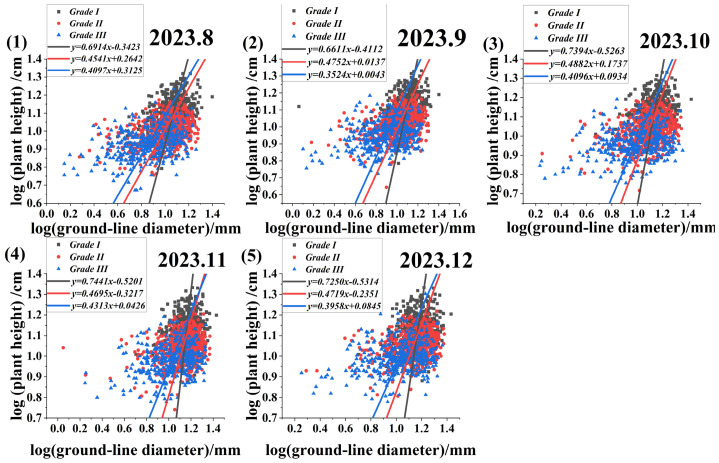
Linear function image of allometric growth equation between height and ground−line diameter of different grades of annual *P. yunnanensis* seedlings on different dates of (**1**) August 2023, (**2**) September 2023, (**3**) October 2023, (**4**) November 2023, (**5**) December 2023.

Figure 5 illustrated the plot of the linear function of the allometric equation for height and ground-line diameter of 2023 from August to December. The 2023 of Grade I, II and III seedlings from August to December also showed a significant linear relationship between ground-line diameter and seedling height, however, the slope and intercept of Grade I seedlings increased rapidly from 0.6914 in August to 0.7441 in December, the growth rate was always the first of the three grades, corresponding to the “seedling height growth per unit ground-line diameter increment” continued to enlarge, and the growth rate showed “high reacceleration”; The slope of Grade II seedlings increased slowly from 0.4541 in August to 0.4882 in October, but decreased to 0.4695–0.4719 in November and December, the slope of Grade III seedlings was always the lowest, which was 0.4097 in August. After rising slightly to 0.4313 in October, it fell back to 0.3958 in December, and the intercept decreased from + 0.3125 to −0.0845 with month. The results showed that the ground-line diameter had the weakest driving force on seedling height, the growth rate was low throughout and there were signs of stall, and the differentiation between individuals was significantly intensified. In general, after autumn, Grade I seedlings established an absolute advantage with a continuous climbing slope and rate, Grade II seedlings lacked power in the late stage, and Grade III seedlings were fully backward and differentiated. The maximum difference in growth efficiency among grades was observed in late autumn.

Therefore, combined with the results from September to 2023 December in 2022, Grade I seedlings showed an allometric growth of “sustained high efficiency-second acceleration” with the highest annual and twice-jump slope from 0.63 to 0.74; The slope of Grade II seedlings was the lowest at 0.24 in early stage, and reached the peak at 0.51 in summer after fast 2023, and then stabilized, forming a “catch-up-platform” rate curve The slope of grade III seedlings was always the lowest between 0.31 and 0.43, and the intercept was high, from positive to negative, showing slow growth and continuous differentiation of “low efficiency-high variation”. In conclusion, the Grade I seedlings had the best growth efficiency, the Grade II seedlings tended to slow down in the late stage, and the Grade III seedlings lagged behind in the whole process. The difference in growth efficiency among grades reached the maximum in summer and autumn of the 2023 season, forming a clear and stable differentiation gradient.

### 3.2. Allometric Growth of Root Length and Root Average Diameter of Seedlings of Different Grades

Table 2 illustrated the root allometric growth of three different grades of seedlings from September 2022 to December 2023. Analysis of the data revealed significant differences in growth patterns among the different grades. Grade I showed allometric growth at most time points, and its slope decreased sharply from 2.849 to −5.798, and the *R*^2^ value was generally low, with a maximum of 0.431. The negative slopes of root growth rate in June and December were −3.225 and −5.798, respectively, which might reflect the 2023 reversal of growth direction or resource allocation. Grade II was dominated by allometric growth, with a relatively stable slope from 1.331 to 3.119, but the *R*^2^ value was low, and the highest was 0.368, reaching 0.698 only in the 2023 September, indicating that the model fitted well during this period. In September, the *R*^2^ of 2023 significantly increased to 0.698, and the slope turned positive to 2.729, which may indicate a change in growth strategy. In general, root allometric growth showed obvious spatial and temporal heterogeneity: Grade I had the most intense dynamics, Grade II was relatively stable but had insufficient explanatory power, and Grade III had a significant optimization of growth pattern in the late stage.

**Figure 6:**
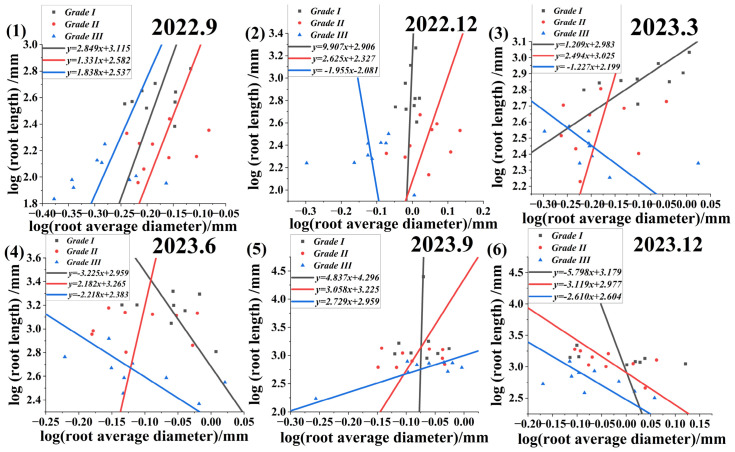
Linear function image of allometric equation between root length and root average diameter of different grades of *P. yunnanensis* seedlings on different dates of (**1**) September 2022, (**2**) December 2022, (**3**) March 2023, (**4**) June 2023, (**5**) September 2023, (**6**) December 2023.

Figure 6 illustrated the allometric trends in root length and root average diameter of different grades of annual *P. yunnanensis* seedlings on different dates, from September to 2023 December 2022, the regression slope of root average diameter to root length showed a dramatic change of “first positive, then negative, and steep downward” with time and grade: the slope of Grade I seedlings declined after reaching a peak of 2.85 in 2022, and the root length of Grade I seedlings declined after reaching a peak of 2.85 in 2022, the 2023 growth rate suddenly turned from −5.80 in June to −3.23 in September and remained around −3.23 in December The slope of Grade II seedlings increased slowly from 2.49 in September 2022 to 2.63 in March 2023, and also turned sharply negative to −3.12 in June, then decreased slightly to −2.22 in September and to −2.61 in December, the slope of Grade III seedlings was always the lowest, only 1.33 in September 2022, and the 2023 rose slightly to 1.23 in March, after a short-term surge to 4.84 in June, the growth rate rapidly turned negative to −2.61 in September, and then dropped to −2.61 in December. The growth rate was depressed for a long time and contracted negatively again after a short-term abnormal surge in summer and autumn, with the most intense individual differentiation. Overall, the root system of the three different grades showed a simultaneous “positive to negative” slope in June 2023, and then the absolute value of the negative slope was in the order of Grade I > Grade II > Grade III. The growth rate also decreased gradually from high to low, and the difference between grades reached the maximum in the summer and autumn of 2023.

Therefore, the allometric growth of root average diameter and root length of Grade I, II and III seedlings showed a distinct trajectory of “first positive and then negative, grade gradient solidification”: the growth rate of root system of Grade I seedlings was “high-speed elongation-rapid stop contraction”, the absolute value of negative value was always the largest, indicating that the root system was the most sensitive to resource redistribution The root growth rate of Grade III seedlings had a long-term depression and accompanied by violent fluctuations, showing the largest individual difference and the worst growth control. In conclusion, Grade I root system had the advantage of “high efficiency-controlled contraction”, Grade II was “medium speed-lag negative turn”, and Grade III was “low efficiency-high variation”, the difference of allometric efficiency among different grades may reach the extreme value in the summer and autumn of the 2023, which provides a clear time window and physiological basis for the management of graded water and fertilizer and root pruning in the later stage.

### 3.3. Analysis of Phenotypic Plasticity Index of Different Grades of Seedlings

**Figure 7:**
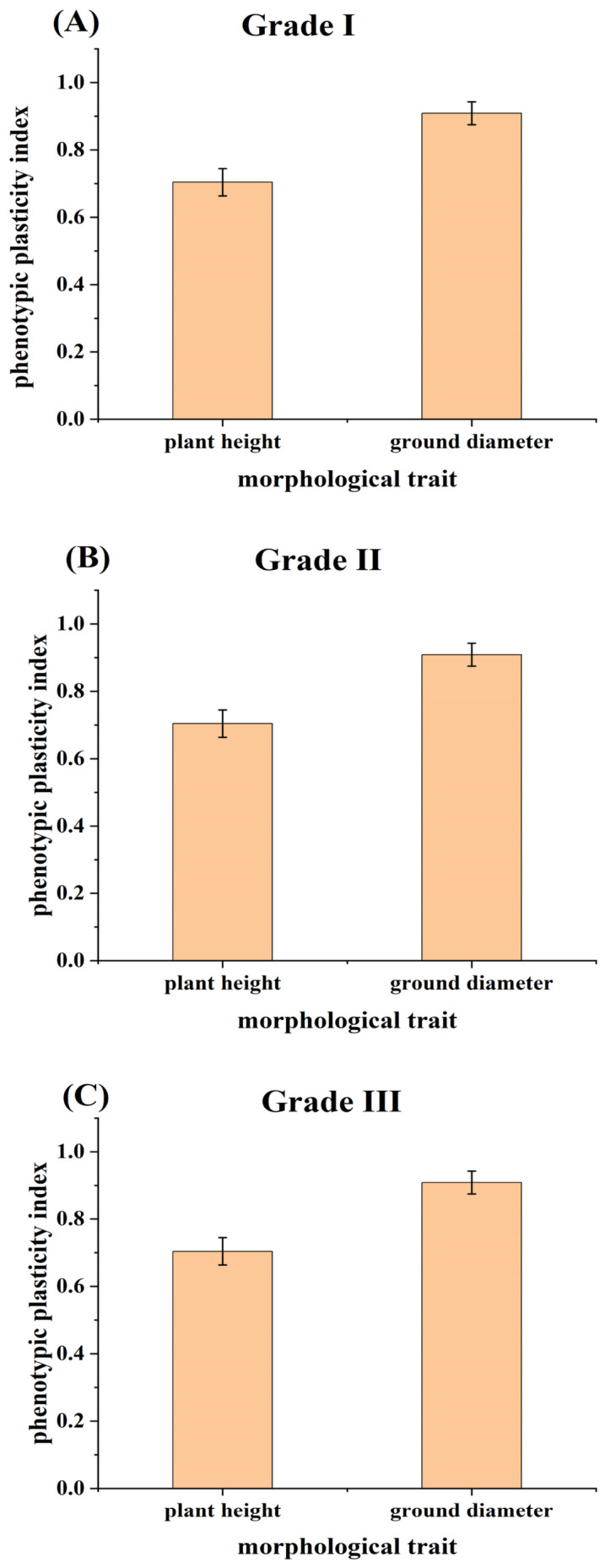
Phenotypic plasticity analysis of grading morphological characteristics of *P. yunnanensis* seedlings.

Figure 7 illustrated the phenotypic plasticity index of seedling height and ground diameter of *P. yunnanensis* seedlings differs clearly among grades. Regarding seedling height plasticity, Grade I seedlings had a plasticity index of 0.6 to 0.7, which is relatively high, indicating a good potential for upward growth. The seedling height plasticity of Grade II seedlings decreased to 0.3 to 0.7, falling within a moderate range, suggesting reduced ability for upward growth and a shift in growth strategy toward other directions. For Grade III seedlings, the numerical value of seedling height plasticity reached 0.6 to 0.8, which seemed high, but this must be understood in combination with absolute growth: Grade III seedlings started with very small initial size and extremely weak overall growth vigor; therefore, this relatively high plasticity index cannot translate into actual height gain, and their ability to respond to environmental changes is very limited.

Regarding ground diameter plasticity, all three grades showed very high plasticity indices. Grade I and Grade II seedlings had a ground diameter plasticity index of about 0.9, and Grade III seedlings also reached 0.9. This indicated that regardless of seedling quality, thickening of ground diameter appears to be a universally prioritized trait for *P. yunnanensis* seedlings. However, the high ground diameter plasticity of Grade III seedlings needed careful interpretation: because their initial ground diameter was very low, even with a high plasticity index, the absolute amount of thickening was still far less than that of Grade I and Grade II seedlings. Grade I seedlings can achieve rapid expansion in both seedling height and ground diameter simultaneously, developing a growth strategy of expanding both upward and outward. Grade II seedlings relied more on ground diameter thickening, with relatively conservative height growth. Grade III seedlings were limited by their low starting point and insufficient overall vigor despite not having low plasticity indices; they cannot convert their plasticity advantage into effective morphological growth, nor can they compensate for their inherent lack of growth potential.

In summary, the phenotypic plasticity indices of seedling height and ground diameter do not function in isolation but are closely related to initial seedling quality. Grade I seedlings achieve both high plasticity and high absolute growth in both traits. Grade II seedlings display a moderate plasticity strategy centered on ground diameter. Grade III seedlings fall into a situation of high plasticity indices but low realized growth.

## 4. Discussion

### 4.1. Factors Affecting the Allometric Growth Trend of Seedling Height and Ground-Line Diameter

Seedling morphology refers to the shape or structure of seedlings, which is easy to measure and can predict the effect of seedling afforestation to a certain extent [36]. At home and abroad, the grade standards of seedling quality are mostly focused on the more intuitive morphological indicators comprising key morphological traits like seedling height and ground-line diameter [37]. The advantages are simple operation in the production process, can be measured by simple instruments, and the evaluation process takes less time; the results are straightforward [15]. Researchers have used principal component analysis to analyze some morphological indicators of *Fokienia hodginsii* container seedlings, and found that overall seedling quality is usefully indicated by measurements of seedling height and ground-line diameter [38]. In addition, the researchers investigated the ground-line diameter and seedling height growth of *Juglans regia* seedlings, and found that the ground-line diameter growth increased with the increase of seedling height growth over time [39]. Under local conditions, *P. yunnanensis* seedlings stopped growing from late December to early February due to winter dormancy [40]. In January, their ground diameter, height, and biomass showed almost no change, so their growth pattern was very similar to that in December and February [41]. Therefore, according to the growth rhythm of *P. yunnanensis*, the growth in January can be ignored. At the same time, according to the analysis of allometric growth of different grades of annual *P. yunnanensis* seedlings under different dates, it was also found that the growth of seedling height and ground-line diameter of different grades of seedlings was significantly increased.

However, there are many factors affecting seedling height and ground-line diameter. For example, under suitable conditions, the impact of planting density was pronounced on the development of seedling height and ground-line diameter. The researchers found that in good site conditions, the smaller the spacing, the worse the quality, and planting in poor fertility and high latitude, which is the most adverse effect on the growth of seedlings [42]. After investigation, the latitude of Kunming is 25 degrees north latitude, lower than most cities in the country, with less absorption of sunlight than at high latitudes, and the region is more [43]. According to the soil investigation and analysis, the soil in the Kunming area has high organic composition, good soil maturity, moderate fertility, and the average pH value is 6.4 [44]. In addition, for potted plants, each pot organ has a relatively independent growth environment; pots are isolated from each other, so the root systems will not affect each other, allowing seedlings to maintain independent growth conditions [45]. In this study, we conducted a Southwest Forestry University survey in a 25°04′00″ N Kunming nursery, the seedlings of different grades of annual *P. yunnanensis* were placed in a plastic pot with a height of about 13 cm, an inner ground-line diameter of about 14.5 cm, an outer ground-line diameter of about 15.5 cm, and a volume of about 306.9 cm, red soil with PH 6 was added for seedling cultivation. Therefore, the above operations made the growth of annual *P. yunnanensis* seedlings occupy excellent geographical conditions, with sufficient light and suitable soil fertility. At the same time, the seedlings of annual *P. yunnanensis* were also used as potted plants to solve and overcome the significant adverse effects of seedling spacing on seedling height and ground-line diameter growth in seedling density. The growth performance in terms of seedling height and ground-line diameter was satisfactory across different seedling grades and assessment dates.

Annual *P. yunnanensis* seedlings all exhibit highly significant allometric relationships, but the performance of seedlings of different grades and provenances in fitting the height–ground diameter allometric growth model varies [46]. In practical production, Grade I and Grade II seedlings were generally the targets for cultivation, while Grade III seedlings falling below the average can be treated as unqualified. As the seedling quality grade improves, the allometric growth index decreased accordingly, and the allometric growth model changed correspondingly [47]. However, the allometric relationship model between height and ground-line diameter for annual *P. yunnanensis* seedlings shifted from conforming to the stress self-similarity model to not conforming to any model [48]. The reason may be that annual *P. yunnanensis* is in the early stage of growth and development, where various physiological indicators and the development of its mechanical structure are still insufficient to support model construction; at the same time, the allometric relationship of plants may change with age, as allometric growth differs across various developmental stages [48]. Due to differences in structure and function among plant organs, metabolically active tissues and allometric indices undergo a dynamic process of change throughout ontogeny and are also constrained by environmental conditions [49]. Based on the above analysis, it can be seen that there is a correlation between seedling grading and allometric growth analysis, with different seedling quality grades corresponding to different allometric growth indices.

### 4.2. Factors Affecting the Growth and Development of Seedling Roots

Methodological studies on fine root trait sampling indicate that root sampling is difficult and costly, and that a pilot sample size greater than 5 per soil depth increment can reliably estimate the total sample size required. Therefore, although the root sample size in this study was small, the results showed consistent trends and significant differences, which needed to be verified with a larger sample size in the future [50]. Under normal conditions of plant growth, roots are the important tissues to obtain water and nutrients from the soil and maintain the stable growth of plant individuals [51]. It can be concluded that root allometric growth is predominantly governed by the capacity for water and nutrient uptake. The mechanism of water uptake by roots is very complex, and the rate of water uptake by different parts of roots is different [52]. The time and degree of root development, the distribution of roots in soil and the properties of soil affect the water uptake of roots [52]. Because water can be transported in any direction through the root system, there is a redistribution of water [53]. As a result of long-term interaction between plant roots and different soil environments, it can not only moisten the nutrient-rich shallow soil, but also maintain microbial activity and increased plant nutrient uptake [54]. Research was carried out to characterize the water use ecology of *Platycladus orientalis* and *Quercus variabilis* in Beijing’s highlands [55]. Data from the study revealed a shift in plant water uptake to deeper soil sources as water stress intensified, and the absorption and utilization of surface soil water by *P. orientalis* and *Q. variabilis* increased [56]. In addition, the study found that although the root density was higher in the shallow soil, and roots mitigated low shallow soil water content by enhancing water absorption from the deep wetting layer [57]. The researchers studied the water absorption of different grades of *Medicago sativa* roots, and found that the strength of water absorption was closely related to the root average diameter and depth of different grades, the greater the root average diameter and the deeper the root depth, the stronger the water absorption of the higher-grade *Medicago sativa*, thus improving the adaptability to the environment [58]. Combined with the results of our study on the roots of different grades of annual *P. yunnanensis* seedlings under different dates, under certain potting conditions, the content of water in the soil was limited, and the seedlings of annual *P. yunnanensis* mainly used the middle and deep soil water in potting, because the root average diameter of Grade I seedlings was greater than that of Grade II seedlings and greater than Grade III seedlings, the deeper the root system of Grade I seedlings was, the stronger the water absorption capacity was, after the transportation and redistribution of water in the root system, the growth rate of the seedling root system was improved, so the growth rate of the root system of the Grade I seedling was the highest, and the growth rate of the root system of the Grade III seedling was the lowest.

The effect of soil surface erosion on soil consolidation also has an important influence on root growth [59]. Studies have shown that the root system was distributed in the soil surface, the root average diameter was small, the root distribution density was large, and the bearing capacity of the soil can be improved by the anti-tensile force of the root system itself and the anti-shear force formed by the root-soil composite, so as to keep the growth of root system in a stable state [60]. In addition, the longer and deeper the root, the wider the distribution, the stronger the ability of plant roots to consolidate soil, which is conducive to the growth and development of the root system [61]. Other studies have shown that with the increase of growth years, the root length and the root average diameter of the root system are significantly enhanced, indicating that the ability of plant roots to consolidate soil is closely related to the growth time of plants, which reflects obvious time-scale characteristics [62]. Besides, the research on *Corethrodendron fruticosum* var *mongolicum* found that the increase of root average diameter was positively correlated with the increase of ultimate tensile strength of root system, and there were significant differences in different growth periods, which affected the ability of plant roots to consolidate soil, favoring the growth of plant roots [63]. According to our research and analysis of different grades of annual *P. yunnanensis* roots under different dates, with the passage of time, the higher the grade of the seedlings, the greater the root length and the root average diameter of the root system, and the stronger the ultimate tensile resistance of the root system, this can enhance the ability of the root soil, soil water and nutrients fully supply to the roots of seedlings, after transportation and absorption, so that the root growth ability can be improved. Therefore, the ultimate tensile capacity of Grade I seedling roots was the strongest, which made the roots have the strongest soil reinforcement ability, can fully absorb water and nutrients in the soil, and the growth rate of roots can be rapidly increased.

The variation of root morphology also has a great influence on the survival, growth, and development of plants [64]. The researchers studied the roots of *F. hodginsii* seedlings and found that when the seedlings were confined to a limited environment, a large number of roots sprouted, and these roots greatly affected the overall growth of the seedlings, so as to speed up the growth rate of the seedlings [65]. Moreover, some studies used root genetic traits as breeding indicators, which will be of great significance for the study of genetic breeding and production cultivation of economic forests and some special forests [66]. At the same time, some studies have shown that under light shading, the root morphology of seedlings will undergo a certain degree of adaptive changes, and the root surface area will be enhanced, thereby enhancing nutrient absorption [3]. Combined with the results of our research on different grades of seedlings of annual *P. yunnanensis*, we can see that seedlings in pots have a limited growth environment and may germinate more roots under the influence of the external environment and their own genetic factors. The surface level of the root system may also increase, allowing it to absorb more water and nutrients from the soil. At the same time, Grade I seedlings may have a certain advantage in the three grades; the number of roots may be larger than that of Grade II seedlings than Grade III seedlings, and the roots of Grade I seedlings may have a larger surface area, and absorb greater access to soil water and nutrients, thereby stimulating the growth of seedling roots.

### 4.3. The Interaction Between Allometric Growth and Phenotypic Plasticity

Relevant researchers have found that the proportion of substances related to stoichiometric characteristics such as C, N and P in various organs of annual *P. yunnanensis* seedlings will be significantly affected after receiving certain light, it will also affect the growth of seedlings [67]. Therefore, from the analysis of our research results, under different dates, different grades of seedlings of annual *P. yunnanensis* will receive a certain amount of light in the pot, and begin photosynthesis. After that, the content of chemical element in various organs of seedlings will be adjusted, so that the allometric growth trajectory of different grades of seedlings of annual *P. yunnanensis* will be changed. Because the growth rates of different grades of seedlings are different, the phenotypic plasticity index is also different, that is, the higher the grade of seedlings, the faster the allometric growth rate and the higher the phenotypic plasticity index.

Furthermore, the phenotypic plasticity index is an important indicator to measure the adaptation of organisms to environmental changes [68]. Under different environmental conditions, different phenotypic structures of plants respond to environmental selection, in plant growth and reproduction, population survival and maintenance and other functions of individual organs of the population to achieve optimal allocation of biomass investment to adapt to a diverse environment [69]. However, changing the allocation pattern of resources is one of the self-adjusting strategies of plants in response to environmental changes, which regulates the growth and development of plants [70]. For example, studies have shown that plants allocate more resources to their aboveground parts to meet their normal growth needed when water was abundant, and to their underground parts when water was scarce [70]. This was conducive to their access to more water and nutrients from the soil, to ensure that plants in the unsuitable environment can survive [71]. The researchers also studied the phenotypic plasticity index and growth and development memory of *Aegilops tauschii* seedlings under different light conditions and soil water content treatments, and found that the phenotypic plasticity index of the seedling leaves was the highest, the main stem ratio was the smallest, indicating that *A. tauschii* seedlings adapted to changes in light and soil water content mainly by adjusting indicators related to leaf morphological traits [72]. Our investigation into the temporal development of different seedling grades in annual *P. yunnanensis* demonstrated clear differentiation in growth characteristics among the graded groups can obtain relatively sufficient water and light energy by cultivating and growing in pots in a certain space. Therefore, *P. yunnanensis* will allocate more limited resources to the main stem and adjust the seedling height, and ground-line diameter phenotypic plasticity index, making the phenotypic plasticity index of plant height greater than the phenotypic plasticity index of ground-line diameter to adapt to changes in light and soil moisture. In addition, the classification of seedling grades also played a certain role. The higher the seedling grade, the greater the phenotypic plasticity index in the same organ, the faster the allometric growth rate, and the better the growth effect, the greater the contribution rate to the late afforestation effect. In summary, the phenotypic plasticity index of Grade I seedlings was higher than that of Grade II and Grade III seedlings, the allometric growth rate was relatively fast, and the contribution rate of afforestation effect in the later stage was phenotypic plasticity.

Besides, the phenotypic plasticity of plants is also affected by biotic and abiotic factors, such as foragers, competitive plants, light, water, and nutrition [73,74]. From the view of plant allometric growth, it can be divided into active plasticity and passive plasticity [75]. Plant phenotypic plasticity is a concrete expression of interspecific competition, which is formed by many factors, with consideration of environmental conditions on light or water and plant morphological properties [76]. The intensity of competition for light in the aboveground part of plants depends on the light demand characteristics and plasticity of plants [77]. The phenotypic plasticity of plants is influenced by the competition between neighboring plants for water, and the competition between plants for water and nutrients is inseparable [78]. Researchers have found that the same type of plants was found in the same niche, so the competition for light resources was very fierce, and plants with higher plant height had obvious competitive advantages [79]. Because of the asymmetric competition among plants, the species with competitive advantage become more powerful [80]. However, the competitive disadvantage is getting weaker and weaker, and some of them will compensate for their disadvantages through strategies such as vegetative propagation [81]. This is related to the phenotypic plasticity ability of the species itself, which in turn affects the growth and development of the species, so the stronger the competitive advantage, the stronger the phenotypic plasticity ability, the stronger the growth and development ability [81]. From our phenotypic plasticity analysis and allometric analysis of different grades of annual *P. yunnanensis* seedlings at different dates, Grade I, II and III seedlings were grown in certain pots, they can absorb relatively sufficient water and nutrients, but their absorption of light is different. Because of their similar ecological niches, the competition for light resources among them was very fierce. In addition, there will be asymmetric competition among three different grades of seedlings. The height, ground-line diameter and root system of Grade I seedlings were relatively high, which had obvious competitive advantages, and the competitive disadvantages will become smaller and smaller, through the late allometric growth to make up for their own disadvantages. In conclusion, Grade I seedlings had a strong competitive advantage when performing asymmetry to external light with Grade II and III seedlings, enabling access to more resources, and the ability to increase their productivity, the phenotypic plasticity index will be greater than that of the second and third grade seedlings, and eventually the allometric trajectory and growth rate of the seedlings will be faster than those of the second and third grade seedlings, it provided a strong scientific basis for the selection of high-quality seedlings and afforestation benefits in the later stage.

In summary, based on the above discussion and our analysis of the results, there were significant differences in allometric growth and phenotypic plasticity among different quality grades of annual *P. yunnanensis* seedlings. Grade I seedlings showed the strongest performance in height, ground-line diameter, and root development, characterized by rapid growth and strong adaptability. Grade II seedlings exhibited moderate growth, with slower initial growth followed by accelerated development, and relatively stable root growth. Grade III seedlings showed the poorest growth performance; although they had high plasticity and notable root variation, their growth efficiency was low. Therefore, seedling height, ground-line diameter, and root traits were reliable indicators of seedling quality. The differences in growth efficiency among different grades were most pronounced in summer and autumn, providing a useful basis for graded seedling management.

## 5. Conclusions

The allometric growth and phenotypic plasticity of annual *P. yunnanensis* seedlings differed significantly among grades. Grade I seedlings grew the best in height, ground diameter, and roots. Their allometric slope rose from 0.43 to 0.74, and their root slope turns from positive to negative with the largest absolute value. This showed a strategy of fast growth and quick adjustment. Their plasticity index was 0.6 to 0.7 for height and about 0.9 for ground-line diameter. Grade II seedlings have an allometric slope that rises from 0.24 to 0.51 and then stabilizes. Their root slope was relatively stable. Their growth was slow at first and then speeds up. Their height plasticity was 0.3 to 0.7, and ground diameter plasticity was also about 0.9. Grade III seedlings had the lowest allometric slope between 0.31 and 0.43. Their roots changed a lot. Their height plasticity reached 0.6 to 0.8, but they started very small. Their ground diameter plasticity was as high as 0.9, yet they still cannot thicken well. Therefore, their actual growth was the worst, showing low efficiency and high variation. Seedling height, ground diameter, and root traits were good signs of seedling quality. The difference in growth efficiency among grades was largest in summer and autumn, which helped us manage seedlings by grade. In the future, seedlings of *P. yunnanensis* can be graded early based on traits such as height and ground diameter. During summer and autumn, when growth differences among grades are largest, differential water and fertilizer management can be applied to each grade. This approach will increase the proportion of high-quality seedlings, reduce waste from weak seedlings, and improve survival rate and stand uniformity after planting.

## Figures and Tables

**Table 1:** Allometric growth analysis of plant height and ground-line diameter of different grades of annual *P. yunnanensis* seedlings on different dates (notes: relationships were categorized T as allometric (A) or isometric (I)).

Date	Grade	*n*	*R* ^2^	Slope	Intercepts	*p*	Type
September 2022	I	468	0.201	0.4337	0.7289	0.000	A
	II	569	0.059	0.2372	0.6741	0.000	A
	III	411	0.012	0.5309	0.4946	0.000	A
October 2022	I	468	0.195	0.4749	0.6967	0.000	A
	II	569	0.142	0.3469	0.6572	0.000	A
	III	411	0.066	0.6264	0.4950	0.000	A
November 2022	I	468	0.218	0.4591	0.6773	0.000	A
	II	569	0.261	0.3301	0.6720	0.000	A
	III	411	0.181	0.4548	0.5744	0.000	A
December 2022	I	468	0.243	0.4634	0.6657	0.000	A
	II	569	0.253	0.3107	0.6838	0.000	A
	III	411	0.167	0.4335	0.5883	0.000	A
February 2023	I	468	0.139	0.4723	0.5406	0.000	A
	II	569	0.322	0.3467	0.6417	0.000	A
	III	411	0.271	0.6261	0.5794	0.000	A
March 2023	I	468	0.181	0.4877	0.6132	0.000	A
	II	569	0.315	0.3657	0.6557	0.000	A
	III	411	0.263	0.4181	0.6069	0.000	A
April 2023	I	468	0.175	0.5446	0.5628	0.000	A
	II	569	0.301	0.4077	0.6277	0.000	A
	III	411	0.274	0.4623	0.5757	0.000	A
May 2023	I	468	0.149	0.5871	0.5258	0.000	A
	II	569	0.139	0.5057	0.5533	0.000	A
	III	411	0.243	0.4617	0.5710	0.000	A
June 2023	I	468	0.121	0.6223	0.4902	0.000	A
	II	569	0.247	0.4415	0.5869	0.000	A
	III	411	0.209	0.4394	0.5698	0.000	A
July 2023	I	468	0.100	0.6831	0.4017	0.000	A
	II	569	0.173	0.4511	0.5566	0.000	A
	III	411	0.147	0.4212	0.5614	0.000	A
August 2023	I	468	0.079	0.6914	0.3650	0.000	A
	II	569	0.152	0.4541	0.5486	0.000	A
	III	411	0.128	0.4097	0.5794	0.000	A
September 2023	I	468	0.055	0.6611	0.3954	0.000	A
	II	569	0.126	0.4752	0.5309	0.000	A
	III	411	0.081	0.3524	0.6352	0.000	A
October 2023	I	468	0.042	0.7394	0.2870	0.000	A
	II	569	0.082	0.4882	0.4972	0.000	A
	III	411	0.067	0.4096	0.5586	0.000	A
November 2023	I	468	0.026	0.7441	0.2688	0.000	A
	II	569	0.069	0.4695	0.5095	0.000	A
	III	411	0.082	0.4131	0.5522	0.000	A
December 2023	I	468	0.031	0.7250	0.2887	0.000	A
	II	569	0.084	0.4719	0.5140	0.000	A
	III	411	0.031	0.3958	0.5833	0.000	A

**Table 2:** Allometric growth analysis of root length and root average diameter of different grades of annual *P. yunnanensis* seedlings on different dates (notes: relationships were categorized T as allometric (A) or isometric (I)).

Date	Grade	*n*	*R* ^2^	Slope	Intercepts	*p*	Type
September 2022	I	9	0.068	2.849	3.115	0.011	A
	II	9	0.000	1.331	2.582	0.468	I
	III	9	0.043	1.838	2.537	0.124	I
December 2022	I	9	0.015	9.907	2.906	0.000	A
	II	9	0.080	2.625	2.327	0.017	A
	III	9	0.011	−1.955	2.081	0.096	I
March 2023	I	9	0.371	1.209	2.983	0.545	I
	II	9	0.062	2.494	3.025	0.024	A
	III	9	0.309	−1.227	2.199	0.533	I
June 2023	I	9	0.431	−3.225	2.959	0.001	A
	II	9	0.008	2.182	3.265	0.056	I
	III	9	0.390	−2.218	2.383	0.020	A
September 2023	I	9	0.000	4.847	4.296	0.000	A
	II	9	0.036	3.058	3.225	0.008	A
	III	9	0.698	2.729	2.959	0.001	A
December 2023	I	9	0.078	−5.798	3.179	0.000	A
	II	9	0.368	−3.119	2.977	0.002	A
	III	9	0.325	−2.610	2.604	0.009	A

## Data Availability

All data generated or analyzed during this study are included in this article. All data generated or analyzed during this study are available from the first author or request.
